# Assessing the value of a real-time electronic screening algorithm for early detection of severe sepsis in the emergency department

**DOI:** 10.1186/cc14025

**Published:** 2014-12-03

**Authors:** P Martin-Rico, A Valdivia-Perez, JM Lacalle-Martinez, J Chorda-Ribelles, MD Marco-Lattur, N Lozano-Cortell, M Jordan-Lluch, E Mateo-Sanchis, R Andres-Navarro, P Olcina-Lloret, JD Garcia-Pedro, O Esparcias-Rodriguez, J Magraner-Egea, A Barcelo-Lopez

**Affiliations:** 1 Internal Medicine (Infectious D. Unit), Hospital de Denia, Denia Alicante, Spain; 2 Preventive Medicine, Hospital de Denia, Denia Alicante, Spain; 3 IT Department, Hospital de Denia, Denia Alicante, Spain; 4 Microbiology Department, Hospital de Denia, Denia Alicante, Spain; 5 Emergency Department, Hospital de Denia, Denia Alicante, Spain

## Introduction

In severe sepsis/septic shock (SS/SS), early recognition and timely implementation of treatment is critical for survival, and this could be electronically supported. We assess the value of an electronic automatic algorithm based on EMR data as a screening tool for early detection of sepsis.

## Methods

Our multiprofessional sepsis team (clinicians and IT engineers) developed an electronic algorithm using data from our EMR (Cerner Millennium platform) aimed at the automatic, real-time recognition of two or more systemic inflammatory response syndrome components + one or more organ failure parameter (according to sepsis definition) in every patient attended in the ER. The firing of this sepsis rule issues an alert to the responsible clinician to confirm an infectious etiology and opens an electronic standardized order set according to sepsis bundles. The alert database (from its start in May 2013 to December 2013) was cross-matched with the minimum basic data set for urgent admissions (>14 years) during this same period. We selected, based on an *ad hoc *syntaxes, those admissions due to sepsis. Medical records were manually reviewed for confirmation of SS/SS. We assessed sensibility, specificity, negative and positive predictive value of the electronic rule, considering the confirmed clinical diagnosis at discharge as the gold standard.

## Results

In total, 37,323 patients were seen in the ER, 5,657 emergency admissions took place and 178 were due to SS/SS. Alert fired in 1,190 (3.2%) total emergencies and in 754 emergency admissions (13.3%). Data analysis after alert implementation identifies a global sensitivity of 80%, which improved after the first 2 months of transition. In the last 6 months (consolidated period) it was between 85 and 90%. Global specificity 89%, NPV of 99% and PPV of 19% for a global prevalence at admission of 3.2 cases/100 (Table 1). The mean door-to-alert time was 167 minutes (SD 193). Median door to needle (antibiotic) time in the alert-sepsis group was 259 minutes vs. 417 minutes in the group where alert did not fire. In the alert + order set group, antibiotic treatment was adequate in 70% vs. 35% in the nonalert sepsis group (Fisher's exact = 0.001). Overall mortality among alert-fired admissions not due to sepsis was 15% (RR for death during admission: 5.33, 95% CI: 3.73 to 7.59; *P *< 0.0001).

**Table 1:** Prevalence of SS/SS upon admission and sensitivity, specificity, and predictive values of the sepsis rule

Month	Prevalence	Sensitivity	Specificity	PPV	NPV
May	3.1 (1.8 to 4.5)	72.7 (51.8 to 93.6)	88.3 (85.8 to 90.7)	16.7 (8.7 to 24.6)	99.0 (98.1 to 99.9)
June	3.0 (1.6 to 4.3)	55.0 (30.7 to 79.3)	89.2 (86.7 to 91.6)	13.4 (5.4 to 21.4)	98.5 (97.4 to 99.6)
July	3.0 (1.7 to 4.3)	86.4 (69.8 to 100)	89.8 (87.5 to 92.1)	20.7 (11.8 to 29.5)	99.5 (89.9 to 100)
August	1.8 (0.8 to 2.8)	78.6 (53.5 to 100)	87.8 (85.4 to 90.2)	10.7 (4.2 to 17.1)	99.6 (99.0 to 100)
September	2.2 (1.0 to 3.4)	86.7 (66.1 to 100)	88.0 (85.4 to 90.5)	14.0 (6.4 to 21.6)	99.7 (99.1 to 100)
October	4.3 (2.7 to 5.9)	82.8 (67.3 to 98.2)	90.6 (88.3 to 92.9)	28.2 (18.1 to 38.4)	99.2 (98.3 to 100)
November	5.4 (3.5 to 7.2)	84.9 (71.1 to 98.6)	88.9 (86.2 to 91.5)	30.1 (20.3 to 40.0)	99.1 (98.1 to 100)
December	2.9 (1.6 to 4.2)	90.5 (75.5 to 100)	88.4 (85.9 to 90.8)	19.0 (10.8 to 27.2)	99.7 (99.1 to 100)
Total	3.2 (2.7 to 3.6)	80.1 (73.9 to 86.3)	88.8 (88.0 to 89.7)	19.0 (16.1 to 21.8)	99.3 (99.0 to 99.5)

Results of each period are indicated in percentages with confidence intervals at 95%.

## Conclusion

This pioneering sepsis algorithm identified 80% of SS/SS. The alert prompted adequate antibiotic treatment and shortened door to needle time from 7 to 4 hours. Furthermore, in patients without sepsis the algorithm identifies a poor-prognosis subset (RR for death 4.75 (95% CI: 3.72 to 6.07).

